# A Finite Element Analysis Study of Edentulous Model with Complete Denture to Simulate Masticatory Movement

**DOI:** 10.3390/bioengineering11040336

**Published:** 2024-03-29

**Authors:** Jeong-Hyeon Lee, Jeong-Hee Seo, Shin-Wook Park, Won-Gi Kim, Tae-Gon Jung, Sung-Jae Lee

**Affiliations:** 1 Medical Device Research and Development Center, DENTIS Co., Ltd., Daegu 41065, Republic of Korea; ll6089@dentis.co.kr (J.-H.L.); sjh00@dentis.co.kr (J.-H.S.); psw0315@dentis.co.kr (S.-W.P.); 2 Department of Biomedical Engineering, Inje University, Gimhae-si 50834, Republic of Korea; 3 Department of Dental Technology, Daegu Health College, Daegu 41453, Republic of Korea; wgkim@dhc.ac.kr; 4 Medical Device Development Center, Osong Medical Innovation Foundation, Cheongju-si 28160, Republic of Korea

**Keywords:** complete denture, masticatory movement, edentulous, finite element analysis

## Abstract

The purposes of this study are to establish and validate a finite element (FE) model using finite element analysis methods and to identify optimal loading conditions to simulate masticatory movement. A three-dimensional FE model of the maxillary and mandibular cortical bone, cancellous bone, and gingiva was constructed based on edentulous cone-beam-computed tomography data. Dental computer-aided design software was used to design the denture base and artificial teeth to produce a complete denture. Mesh convergence was performed to derive the optimal mesh size, and validation was conducted through comparison with mechanical test results. The mandible was rotated step-by-step to induce movements similar to actual mastication. Results showed that there was less than a 6% difference between the mechanical test and the alveolar bone-complete denture. It opened 10° as set in the first stage, confirming that the mouth closed 7° in the second stage. Occlusal contact occurred between the upper and lower artificial teeth as the mouth closed the remaining angle of 3° in the third stage while activating the masseter muscle. These results indicate that the FE model and masticatory loading conditions developed in this study can be applied to analyze biomechanical effects according to the wearing of dentures with various design elements applied.

## 1. Introduction

Edentulous is a condition in which all teeth are lost due to factors such as aging, trauma, periodontal disease, or osteoporosis. This condition renders impossible certain functions such as mastication and pronunciation and contributes to an elderly facial appearance [1,2]. The prostheses used in individuals with no remaining teeth are termed complete dentures. These prostheses gain retention through soft tissues such as the gingiva. Complete dentures comprise a denture base and artificial teeth, known for their role in supplementing mastication, pronunciation, function, and facial appearance [3]. The shape of the denture base can be categorized into the inner surface touching the gingiva (basal surface), edge part (border), and side face of the denture (polished surface), while artificial teeth are known for their occlusal table where the teeth touch each other [4].

The side effects of wearing complete dentures include oral pain, stomatitis, decreased retention, and fracture which have been associated with the thickness of the denture base and the length of its border [5,6,7,8,9,10]. As there are limitations in using complete dentures made with various design elements by one patient to analyze the comfort and durability of complete dentures, research is currently being conducted using a computer simulation method called finite element analysis [11]. Some studies have analyzed the stress on the alveolar bone consisting of cortical and cancellous bone, the gingiva, and the complete dentures, depending on the type and material of the denture, to predict the wearer’s comfort or the durability of the complete dentures. Many studies have investigated the effects of wearing complete dentures on edentulous alveolar bones and applying occlusal load, but there is a lack of research being conducted in the same pattern as actual mastication. Through finite element analysis, Cheng et al. [11] evaluated the deformation of the complete dentures in the maxilla following complete dentures fixation and occlusal load application, while Takayama et al. [12] conducted a displacement analysis of the complete dentures by applying a unilateral load to a mandibular model with fixed complete dentures. Shrivastava et al. [13] studied the effects on the alveolar bone and gingiva of different materials used as liners which act as an adhesive between the gingiva and complete dentures. Żmudzki et al. conducted an analysis of whether the pressure generated when wearing complete dentures exceeded the patient’s average pressure pain threshold [14]. However, these studies represented occlusion by simply applying compression or shear loads to parts of artificial teeth. Takayama, Shrivastava, and Żmudzki simplified some model (cortical, cancellous, complete denture etc.) shapes for easier implementation [11,12,13,14]. In the clinic, the mandible is rotated based on condyle for mastication movement, however there are no studies reflecting this clinical environment.

As such, this study aimed to present loading conditions for simulating actual mastication movement after constructing and validating three-dimensional finite element (FE) models of alveolar bone-gingiva and complete denture for use in biomechanical research of complete dentures.

## 2. Materials and Methods

In this study, the maxillary and mandibular cortical bone model constructed by Jung et al. was modified as an edentulous model excluding the teeth [15].

### 2.1. Construction of Edentulous Alveolar Bone-Gingiva and Complete Denture FE Model

**Figure 1:**
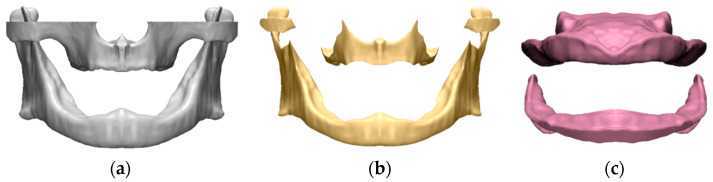
FE model of edentulous alveolar bone and gingiva–alveolar bone, (**a**) cortical bone, (**b**) cancellous bone, and (**c**) gingiva.

Based on previous studies, the modeling program 3-matic (v24.0, Materialise NV, Leuven, Belgium) was used to construct the cancellous bone and gingiva by offsetting 2 mm inward direction and offsetting 2 mm outer direction of cortical bone [16] (Figure 1).

**Figure 2:**
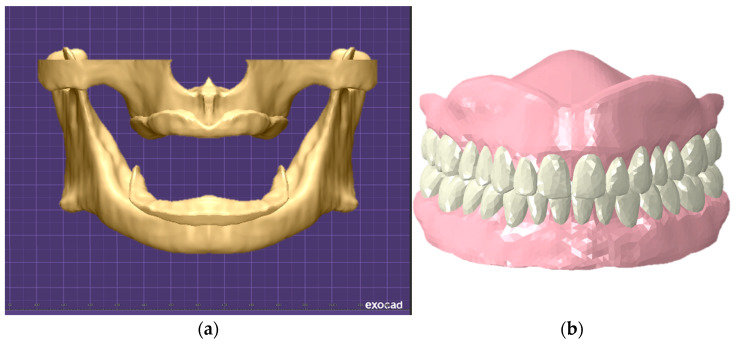
Three-dimensional complete denture model construction; (**a**) simulation of exocad, and (**b**) three-dimensional complete denture model.

Considering the shape of the previously constructed edentulous alveolar bone and gingiva model, step-by-step from the location of teeth and alveolar ridge to the size and shape of the artificial teeth were selected using the dental computer-aided design (CAD) software exocad (v3.1, exocad GmbH, Darmstadt, Germany). In the case of the denture base, it was designed with a thickness of 3 mm and a border length of 0 mm, which is in contact with the border part of edentulous patient. It was then converted into a three-dimensional model (Figure 2).

To confirm whether mastication is possible when there is food in the mouth after complete denture attachment, blocks that fit the size of upper and lower artificial teeth were made using SolidWorks (v2019, Dassault Systèmes SOLIDWORKS Corp., Waltham, MA, USA), a three-dimensional design software. Since the shape and form of food change during mastication, it is difficult to obtain its material properties. Hence, the rubber material properties were applied [17] (Table 1).

The FE model was configured into a tetrahedral element consisting of four nodes for mesh shape using commercial FE software Abaqus (CAE v6.14, Dassault Systèmes Corp., Providence, RI, USA). The contact conditions between cancellous bone and cortical bone, cortical bone and gingiva, gingiva and denture base of the complete denture, and denture base and artificial were set as tie contact assuming complete fusion [18]. The material properties of each model were set with references to the related literature [19,20,21,22] (Table 1). The masseter muscle was constructed using wire based on anatomical shape, and the muscle was contracted during mastication to rose the mandible and the lower artificial teeth to contact with the upper artificial teeth [23,24].

**Table 1:** Material properties of FE models.

Component		Elastic Modulus (MPa)	Poisson’s Ratio	References
Alveolar bone	Cortical bone	18,000	0.31	[19]
	Cancellous bone	500	0.30	
Gingiva		3	0.30	[20]
Artificial teeth		3000	0.35	[21]
Denture base		4500	0.35	[22]
Block (rubber)		4	0.45	[17]

### 2.2. Validation of the Constructed FE Model

#### 2.2.1. Mesh Convergence

**Figure 3:**
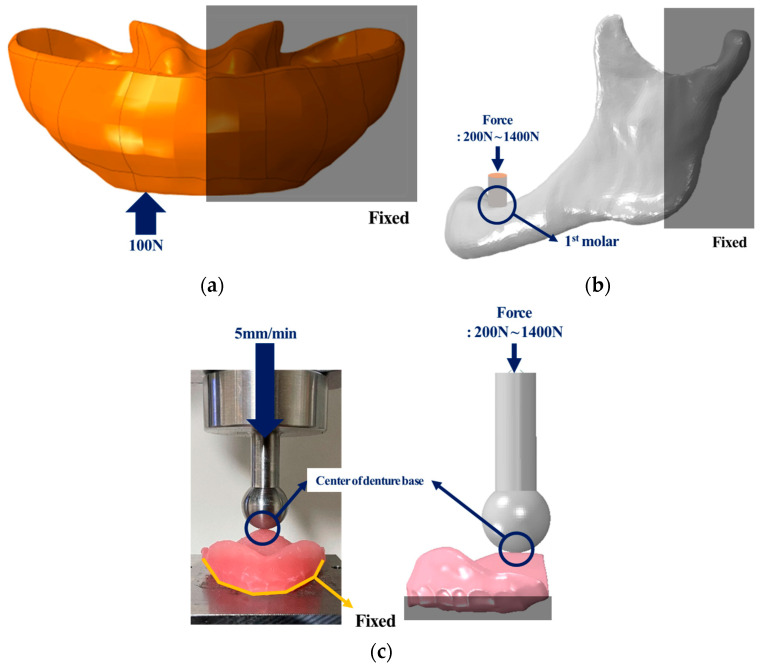
Mesh convergence and validation setup of three-dimensional FE model. (**a**) Mesh convergence, (**b**) validation of edentulous alveolar bone, and (**c**) validation of complete denture.

Mesh convergence was conducted to maintain a balance between the accuracy of results and the analysis time, and to determine the optimal mesh size. The denture base model, a main design element of the complete denture, was utilized for this purpose. The maxillary denture base, which fractures more frequently than the mandible, was selected as the model, and teeth shapes were excluded from the previously constructed denture base model to prevent load concentration due to thin structures between artificial teeth by referring to related previous studies [7]. After constraining movement in all directions on half of the denture base, 100 N, which is the maximum contact force load generated during mastication, was applied to the occlusal surface of the canines, and confirmed the Peak von Mises stress (PVMS) results of the denture base according to mesh size [25] (Figure 3a).

#### 2.2.2. Edentulous Alveolar Bone Model

To validate the constructed edentulous alveolar bone model, compared with the experimental results performed by Sittitavornwong et al. [26]. Sittitavornwong et al. performed a static compression experiment by fixing the left mandibular condyle and posterior mandibular ramus of a cadaver, which the gingiva removed, with cement and then placing a jig on the first molar. Using the same experimental setup as Sittitavornwong et al., only the left alveolar bone model was used and the mandibular condyle area was constrained in all directions in the FE environment. After placing the jig model produced through SolidWorks on the first molar area, a total load of 1400 N was applied to the upper surface of the jig in steps of 200 N [26] (Figure 3b). After analysis, the stiffness of the alveolar bone was then compared with the results of Sittitavornwong et al. for validation.

#### 2.2.3. Complete Denture Model

Referring to the conditions of static compression experiments on the maxillary denture base from previous research, mechanical experiments and FE analysis were conducted [27,28,29]. To perform mechanical experiments, denture base models (*n* = 6) were printed using a three-dimensional printer (Zenith L2, DENTIS Co., Ltd., Daegu, Republic of Korea). The lower part of the prepared specimen was positioned into contact with the lower jig, and the upper jig was placed at the center of the denture base before conducting a static compression test at 5 mm per minute (5 mm/min) using a universal testing machine (MTS 858, MTS system Corp., Eden Prairie, MN, USA). A three-dimensional jig model was created in SolidWorks based on the shape of the upper jig, and the jig and specimen were positioned under identical conditions as in the mechanical experiment. The lower part of denture base was constrained to prevent movement in all directions, and a total load of 1400 N was applied in steps of 200 N to the upper surface of the jig located in the center of the denture base [27,28,29] (Figure 3c). The stiffness results from the mechanical experiment and analysis were compared for validation.

### 2.3. Loading and Boundary Conditions

**Figure 4:**
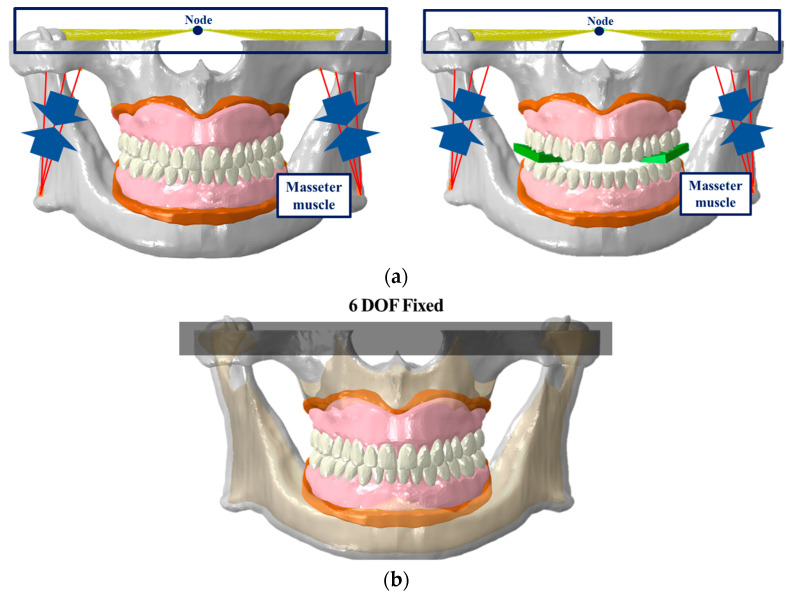
Loading and boundary conditions of masticatory movement. (**a**) Loading conditions without/with food (green substance), and (**b**) boundary condition.

Previous studies on the FE analysis of complete dentures often used simplified models or applied occlusal loads to parts of the artificial teeth, instead of simulating actual masticatory movements by rotating the mandible to contact the maxilla [11,12,13,14]. In the present study, we analyzed three stages to implement actual mastication movements and added a two-stage analysis of food mastication movements to check whether mastication is possible when food is located (Figure 4a). The mastication movement with a complete denture attached to the edentulous alveolar bone and gingiva model was set opening the mouth in the first stage and closing the mouth in the second and third stages. In contrast, food mastication was analyzed as closing the mouth in the first stage and opening the mouth in the second stage.

**Figure 5:**
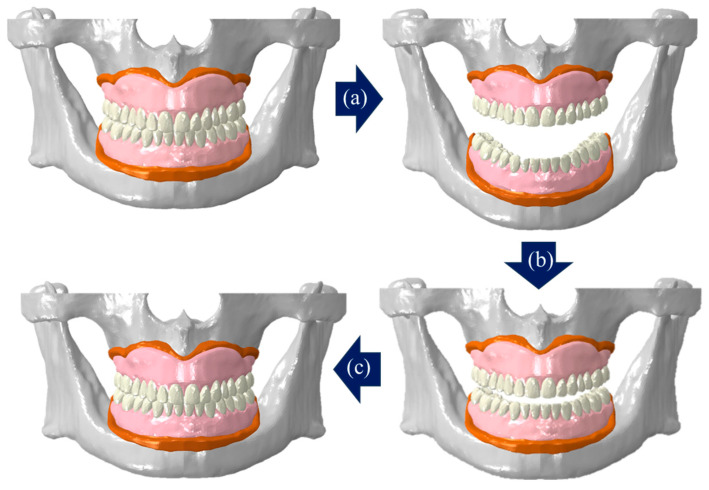
Step of mastication without food. (**a**) Step 1: opening, (**b**) step 2: closing, (**c**) step 3: closing and clenching.

**Figure 6:**
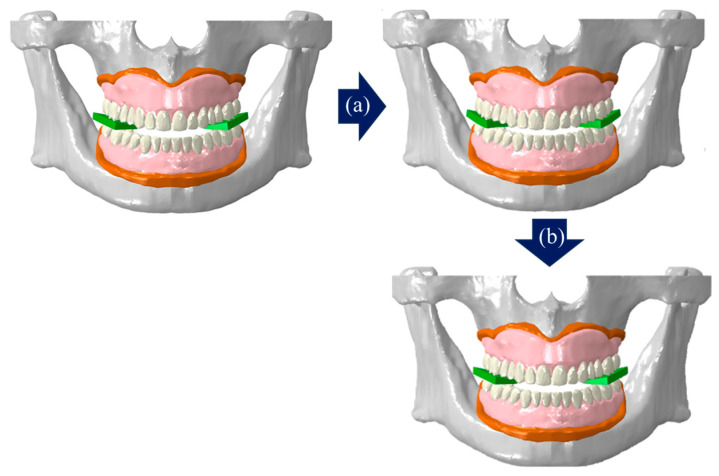
Step of mastication with food. (**a**) Step 1: closing and clenching, (**b**) step 2: opening.

To represent mastication movement, the long axes were implemented on the mandibular condyle using rigid beam elements and a node was then placed in the central part. The node was fixed in translation movement and was set to allow rotation only in the sagittal view. The first stage applied a pure rotational movement of 10° to open the mouth, the second stage involved closing the mouth by 7°, and in the third stage, the mouth was further closed by 3° while activating the masseter muscle load (340 N) [23] (Figure 5). Food mastication movement applied an occlusal load while closing the mouth by 3° in the first stage and represented the opening process by 3° in the second stage (Figure 6). The upper part of the maxillary alveolar bone was constrained in six degrees of freedom (6DOF) to prevent movement in all directions, and the gingiva and complete denture were set in a bonded state using tie contact. Assuming an environment with saliva in the oral cavity, the friction coefficient of the contact surface between the upper and lower artificial teeth and between the artificial teeth and the block was set at 0.2 [30] (Figure 4b). Using Abaqus, contact pressure results were derived to check whether each artificial teeth and block were in contact, predicting mastication movements.

## 3. Results

### 3.1. Validation of the Edentulous Alveolar Bone and Complete Denture FE Model

#### 3.1.1. Mesh Convergence

**Figure 7:**
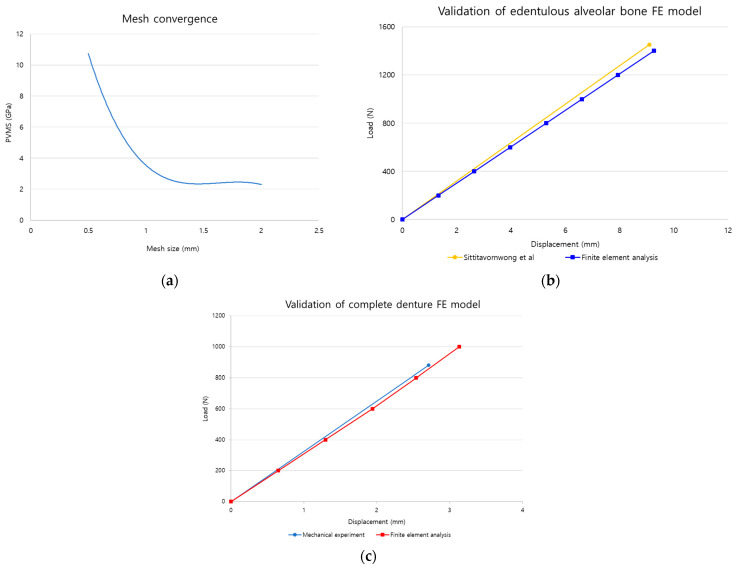
Results of mesh convergence and validation of three-dimensional FE model (graph). (**a**) Mesh convergence, (**b**) validation of edentulous alveolar bone, and (**c**) validation of complete denture.

The mesh size was set to range from a minimum of 0.4 mm to a maximum of 2 mm for analysis, and each denture base’s PVMS result was compared with adjacent mesh results. The comparison showed a maximum difference of 31% and a minimum of 9% before 1.1 mm where the graph started flattening out, while differences were within 5% after 1.2 mm, leading to the application of a 1.2 mm mesh size in this study (Figure 7a).

#### 3.1.2. Edentulous Alveolar Bone Model

After constructing the edentulous alveolar bone model, we compared the experimental and finite element analysis results obtained by Sittitavornwong et al. [26] to validate the validity regarding the use of FE analysis. From the results of Sittitavornwong et al., the average of six load-displacement values corresponding to the edentulous condition was calculated to determine stiffness. For the analysis, the displacement was checked at each 200 N, 400 N, 600 N, 800 N, 1000 N, and 1200 N to calculate stiffness. The experimental and analysis results showed differences within 6% with values of 159.7 N/mm and 151.1 N/mm, respectively (Figure 7b).

#### 3.1.3. Complete Denture Model

Referring to the experimental conditions of Hedzelk [27], Yu et al. [28], and Jadhav et al. [29], an identical environment was created in actual mechanical experiments and FE analysis. The mechanical experiment was performed to calculate the average value of stiffness from a load-displacement curve after performing a static compression test on six specimens, and the FE analysis results were calculated using the displacement results for each load in the same way as the edentulous alveolar bone model validation methods. The mechanical test and finite element analysis results were 324.9 N/mm and 318.2 N/mm, respectively, confirming a difference of less than 2%, validating the validity of the constructed edentulous alveolar bone model and complete denture FE model in this study (Figure 7c).

### 3.2. Implementation of Mastication Movement

**Figure 8:**
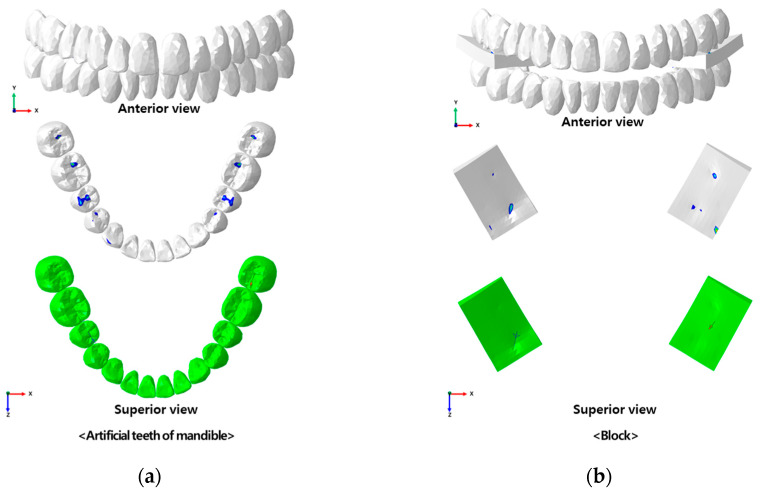
Contact pressure of artificial teeth and block. (**a**) Mastication and (**b**) food mastication.

Our experiments confirmed that when implementing the masticatory movement, the maxilla and mandibular condyle parts were fixed and the mandible is rotated in the sagittal view by a set angle to open and close, and in the third stage, the masseter muscle was activated and the upper and lower artificial teeth came into contact for mastication and occlusion. Additionally, during the implementation of food mastication movements, no errors occurred in the analysis at the first stage, and the block was masticated. Overall, contact between the upper and lower artificial teeth and between the artificial teeth and the blocks from the buccal area to the labial direction was confirmed during both mastication and food mastication analysis (Figure 8).

## 4. Discussion

The edentulous patients wore the prosthesis (called complete denture) to improve mastication, pronunciation, and contribute to elderly facial appearance. As the denture base of a complete denture thickness becomes thicker, absorption of the remaining alveolar ridge may accelerate and cause the denture to fall out due to inconsistency with the inner shape of the denture base. On the other hand, as the denture base thickness becomes thinner, the possibility of complete denture fracture may increase. If the border length of the denture base is overextended, pain and stomatitis may occur. If border length is short, the border area of the denture base may have lower negative pressure and the retention. Depending on the design elements of the complete denture, it can provide comfort or discomfort to the patients and related to the durability of the complete denture. There were some studies that intact alveolar bone or complete denture model and reflected actual masticatory movements. This study aimed to implement actual masticatory movement environment after attempted to construct and validate a FE model of an actual edentulous alveolar bone-gingiva and complete denture.

As mesh size decreases, accuracy increases, however the analysis time also increases, so appropriate balance is needed. It was this need that led us to perform mesh convergence. In this study, after compared adjacent results for each mesh size, a size within 5% of the adjacent results was selected, demonstrating the feasibility of applying the FE model constructed in this study.

Previous studies excluded some models or material properties without conducting validation for those models. In this study, there were no results regarding validation for the developed edentulous alveolar bone-gingiva and complete denture model, making direct comparison impossible. The edentulous alveolar bone model was validated using the method and results comparison of static compression experiments on cadavers [26]. The complete denture model was validated after performing each mechanical experiment and FE analysis under an identical environment based on research that performed mechanical experiments using specimens printed with a three-dimensional printer [27,28,29]. Similar trends between each mechanical experiment and analysis results showed applicability for constructed edentulous alveolar bone-gingiva and the complete denture model in this study.

Unlike the studies by Cheng et al. [11], Takayama et al. [12], Shrivastava et al. [13], and Żmudzki et al. [14], when the loading conditions of this study were applied, the results were not concentrated only on the loaded areas, but moved from the buccal towards the labial direction. It was confirmed that this was similar to the actual mastication patterns [31].

In this study, to implement a mastication environment after attaching actual complete dentures, we activated mandibular rotation and masseter muscle referring to the analytical methods and conditions published by Christensen et al. [23] and Ortún–Terrazas et al. [30]. However, the study only implemented simple mastication and food with low hardness mastication by adjusting the angle, which does not reflect clinical conditions such as clenching, jiggling, or grinding. Future research should analyze the biomechanical effects of complete dentures through the stress at gingiva and PVMS of complete denture, considering oral movements reflected in actual clinical environments by adjusting detailed mastication angles or muscle load control or addition of muscle models [32].

## 5. Conclusions

Our experiments implemented masticatory movements through finite element analysis after constructing maxillary and mandibular alveolar bone-gingiva and complete denture models. By performing mesh convergence, we were further able to determine the optimal mesh size for application. Furthermore, by conducting the analysis in an environment similar to the mechanical experiment and the results were shown to be within the error range, the reliability of the constructed FE model and research findings is expected to be secure. It is thought that biomechanical analyses considering the actual mastication environment will be possible through the contact pattern between the artificial tooth and the block after masticatory analysis.

## Data Availability

The data used to support the finding of this study are included within the article.
